# Mutagenic Deimmunization of Diphtheria Toxin for Use in Biologic Drug Development

**DOI:** 10.3390/toxins7104067

**Published:** 2015-10-10

**Authors:** Joerg U. Schmohl, Deborah Todhunter, Seung Oh, Daniel A. Vallera

**Affiliations:** 1 University of Minnesota Masonic Cancer Center, Section of Molecular Cancer Therapeutics, Therapeutic Radiology-Radiation Oncology, University of Minnesota, Minneapolis, MN 55423, USA; E-Mails: joerg.schmohl@uni-tuebingen.de (J.U.S.); todhu001@umn.edu (D.T.); ohxxx021@umn.edu (S.O.); 2 Department for Hematology and Oncology, Department of Medicine 2, University Hospital of Tuebingen, Tuebingen 72076, Germany

**Keywords:** deimmunization, diphtheria, toxin, biologic drug, cancer, cancer treatment

## Abstract

Background: Targeted toxins require multiple treatments and therefore must be deimmunized. We report a method of protein deimmunization based on the point mutation of highly hydrophilic R, K, D, E, and Q amino acids on the molecular surface of truncated diphtheria-toxin (DT390). Methods: Based on their surface position derived from an X-ray-crystallographic model, residues were chosen for point mutation that were located in prominent positions on the molecular surface and away from the catalytic site. Mice were immunized with a targeted toxin containing either a mutated DT390 containing seven critical point mutations or the non-mutated parental toxin form. Results: Serum analysis revealed a significant 90% reduction in anti-toxin antibodies in mice immunized with the mutant, but not the parental drug form despite multiple immunizations. The experiment was repeated in a second strain of mice with a different MHC-haplotype to address whether point mutation removed T or B cell epitopes. Findings were identical indicating that B cell epitopes were eliminated from DT. The mutant drug form lost only minimal activity *in vitro* as well as *in vivo*. Conclusion: These findings indicate that this method may be effective for deimmunizing of other proteins and that discovery of a deimmunized form of DT may lead to the development of more effective targeted toxin.

## 1. Introduction

Biological drugs show great promise, but in most cases they are limited because they are recognized as foreign by the human immune system [1]. This is particularly true for bacterial proteins such as diphtheria toxin (DT). DT has shown impressive results as a targeted toxin, but even Federal Drug Administration (FDA) approved DT-based drugs, such as ONTAK (DT-IL-2, Eisai, Co.), have the drawback of eliciting anti-toxin responses on multiple treatment [2]. Furthermore, all of us receive diphtheria, tetanus, and pertussis (DPT) immunization at an early age, virtually guaranteeing an anti-toxin response on multiple treatments with DT-based drugs, a finding that has been born out in phase 1 clinical studies [3]. Because DT targeted toxins (TT) have proven effective in eliciting anti-cancer responses in a myriad of different animal models and in patients, the drugs are considered important and phase 1 clinical trials with DT-based drugs are currently underway [4,5,6]. Thus, pursuit of a deimmunized form of the toxin has been considered a desirable goal.

Onda and Pastan partially deimmunized pseudomonas exotoxin (PE) by identifying B cell epitopes and eliminating them through point mutation [7]. PE has a catalytic region and a binding region and kills by ADP ribosylation of elongation factor 2 (EF-2) irreversibly inhibiting protein synthesis [8]. PE38 is a truncated form of PE devoid of the native binding region. Mice were immunized with PE38 containing immunotoxins. Hybridomas reacting with PE38 were isolated and used to identify seven major conformational epitopes located at specific positions on the protein. These were not diffusely distributed over the entire surface of PE38 enabling them to determine the precise location of most of the epitopes by point mutation and showing that specific monoclonal antibody binding to the selected epitope was abolished or greatly decreased [7]. Immunogenicity was significantly reduced by combining several of the mutations.

DT toxin is a 535 amino acid protein (molecular weight 58.3 kDa). It consists of two functional domains and is a close cousin to PE with an identical mechanism of action (reviewed in [9]). The *c*-terminal B domain binds most eukaryotic cells and is removed, forming DT390, and replaced with ligands to form a targeted toxin. The *n*-terminal A domain contains the catalytic enzyme that also ADP-ribosylates EF-2. Following binding, it is taken up in endocytotic vesicles. The toxin is believed to undergo conformational changes which are required for the translocation of the A chain to the cytosolic compartment.

DTEGF13 is a bispecific ligand directed toxin (BLT) comprised of truncated DT (DT390) devoid of its native binding region (B chain) [10,11,12]. Ligands include IL-13 followed by epidermal growth factor (EGF) added to the same single chain protein. The ligands react with unrelated receptors. EGF is the main ligand of the epidermal growth factor receptor (EGFR), a transmembrane signaling protein from the erbB family [13]. EGFR is highly overexpressed on a range of carcinomas including prostate [11], pancreatic [14], breast [15] and mesothelioma [16] and a focus for targeting antibodies like Cetuximab or tyrosin kinase inhibitors (erlotinib, gefitinib) [17].

IL-13 is a useful pleiotropic lymphokine since its receptor is overexpressed in tumors and most B cells and monocytes [18]. DTEGF13 binds to its target via the EGF and IL-13 ligands, is internalized, and then intoxicates target cells [11]. *In vivo*, it is effective locally and systemically against human prostate, glioblastoma, and pancreatic carcinomas in local and systemic xenograft models [10,11,12]. DTEGF13 generates an anti-diphtheria toxin response when injected *in vivo* and was thus a good choice for these mutation studies.

Based on the PE38 deimmunization strategy of mutating R, K, D, E, and Q, we investigated an alternative and simple approach for toxin deimmunization. We identified highly hydrophilic, amino acids for point mutation based on their position on the surface of the molecule derived from an X-ray crystallographic model and their surface positions away from critical amino acid in the active site. We then sequentially screened for those that underwent minimal activity loss. When we obtained candidate mutants with at least seven mutations, we tested them for their ability to generate anti-toxin IgG antibody when given multiple injections. Despite multiple immunizations, our deimmunized DTEGF13 (dDTEGF13) showed a remarkable reduction in anti-toxin induction when compared to the nonmutated parental form in more than one train of mice were affected by mutation.

## 2. Results

### 2.1. Construction

**Figure 1:**
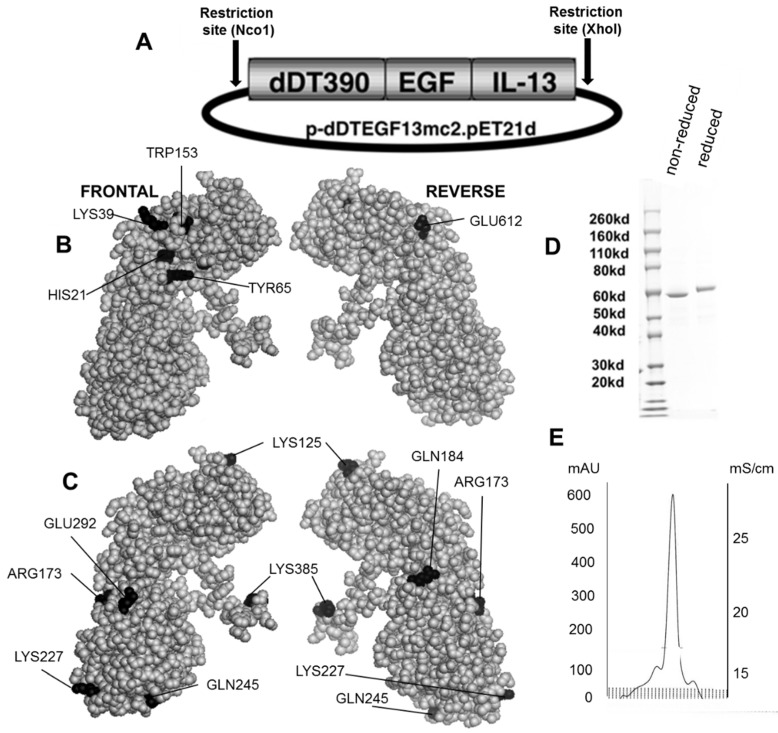
Construction of the plasmid containing the dDTEGF13 gene. (**A**) The pET expression vector containing the dDTEGF13 target gene; (**B**,**C**) The PyMol sphere graphic was generated by downloading the Protein Data Bank [19] X-ray crystallographic structure of DT [20] into the PyMol 3D molecular modeling program [21] Shown is a frontal view of the protein and a 180° reverse view of the molecule; In (**B**), the amino acids associated with ADP-ribosylation (catalytic site) are bl ackened; In (**C**), The amino acids that were mutated for deimmunization are blackened; (**D**) SDS-PAGE gel analysis was performed to confirm the size and purity and stained with Coomasie blue. Photo is grayscale. Lane 1—Molecular weight standards, Lane 2—dDTEGF13 non-reduced, Lane 3—dDTEGF13 reduced. The gel was stained using Coomassie blue; (**E**) A HPLC trace for the purified drug is also shown illustrating mostly a single peak obtained from a TSK3000 size exclusion column. Only the single peak was collected resulting in a >95% purity.

Figure 1B,C shows the dDTEGF13 construction and a PyMol spherical model of X-ray crystallographic structure in both front and reverse (180°) positions. In Figure 1C, the seven mutated amino acids are darkened so their surface position on the molecule can be easily visualized, and Figure 1B shows they are positioned away from the active site. The figure also shows a final SDS-PAGE gel analysis of dDTEGF13 with a purity of greater than 95% as determined by Coomassie blue (Sigma-Aldrich, St. Louis, MO, USA) staining (photo is grayscale, Figure 1D). The procedure was previously reported [11]. Molecular weight size is estimated at 63.8 kDa from molecular weight standards according to known amino acid structure gained from the cloned sequence. With High performance liquid chromatography (Waters Corp., Milford, MA, USA), drug was purified, showing a single peak obtained from a TSK3000 size exclusion column. Only the single peak was collected resulting in a >95% purity (Figure 1E).

### 2.2. Strategy

Although our final deimmunized molecule is shown in Figure 1B,C a number of progenitor mutants were generated and studied. The strategy was to target critical high immunogenicity amino acids. Based on the evaluation of the molecular model derived from the X-ray crystallographic structure, 24 of these residues located in prominent surface positions were identified. A series of eight mutants were generated. Each of the mutants had three point mutations, encompassing 24 of the residues. The choice of which three mutations to include in a given triple mutant was arbitrary. Alanine, serine and glycine substitution were employed. The mutants were quickly screened for activity loss using a standard, highly reproducible proliferation inhibition assay measuring thymidine uptake.

**Figure 2:**
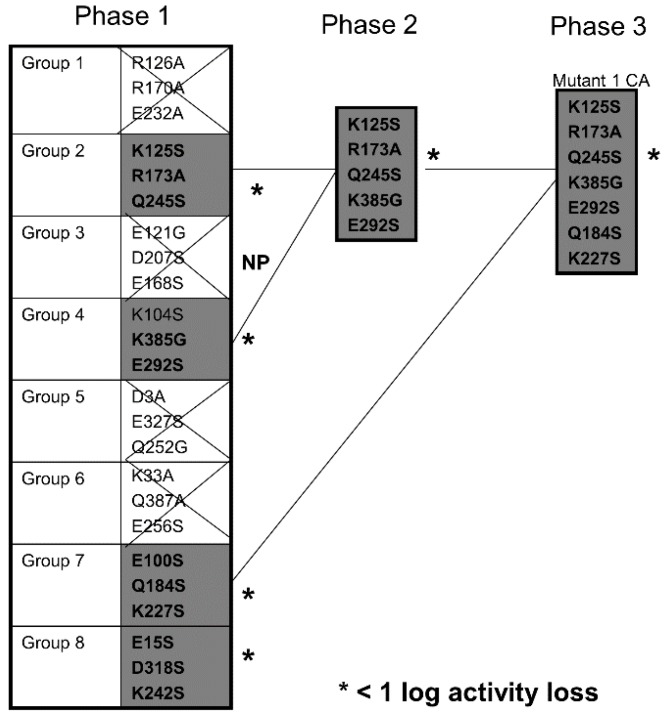
Screening Chart. Chart shows our strategy of producing and screening various DTEGF13 mutants on the path to deimmunization. In phase 1, 8 triple mutants were synthesized and purified. Four of these showed less than a log of activity loss in our *in vitro* screening assay compared to the non-mutated parental control. In phase 2, mutations were combined to generate a quintuple mutant that still had less than a log of activity loss compared to parental. These are marked with an asterisk (*) in the figure. In phase 3, we analyzed a septuplet mutant that still had less than a log of activity loss. Glycine, alanine, and serine substitution were employed.

Figure 2 describes which of the mutants showed minimal activity losses and that they were subsequently combined with other mutants. Choosing mutants that showed less than a log loss, point mutations were combined on the same molecule until we finally obtained DTEGF13 with seven mutations in separate areas of the molecule and less than a log of reduced activity in *in vitro* proliferation assays. This mutant was subsequently tested for its ability to generate an anti-toxin response in two different strains of immunocompetent mice.

### 2.3. Activity of Mutant dDTEGF13

**Figure 3:**
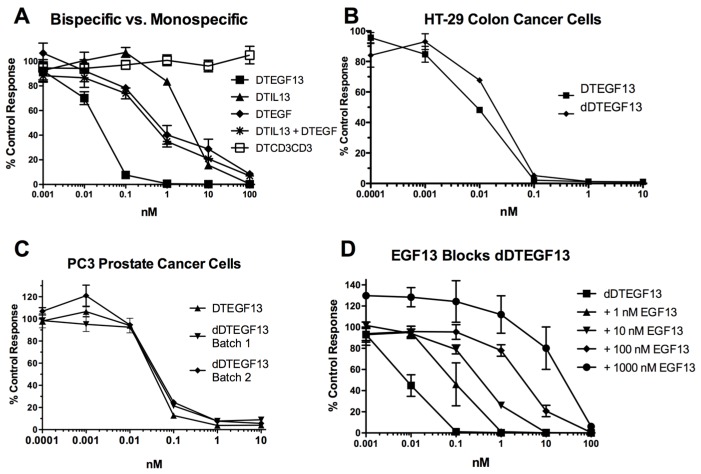
*In vitro* activity of dDTEGF13: (**A**) Bispecific DTEGF13 and its monospecific counterparts were tested and compared for their reactivity against MiaPaCa-2 cells. Proliferation assays were performed by analyzing ^3^H-thymidine uptake after a 72-h incubation with targeted toxins. Data are reported as percent control response. Each data point represents an average of triplicate measures ± SD; (**B**) Deimmunized DTEGF13 and non-mutated parental DTEGF13 were tested and compared for activity against HT-29 colon; and (**C**) PC-3 prostate carcinoma cells in thymidine uptake assays. Two batches are used for reproducibility; (**D**) A blocking assay was performed in which MiaPaca-2 pancreatic cancer cell lines were incubated with an inhibitory dose of dDTEGF13 and then blocked with increasing concentration of EGF13 ligand devoid of toxin. Thymidine uptake was then measured. The non-specific recombinant α-Ly5.2 was included as a negative blocking control.

**Figure 4:**
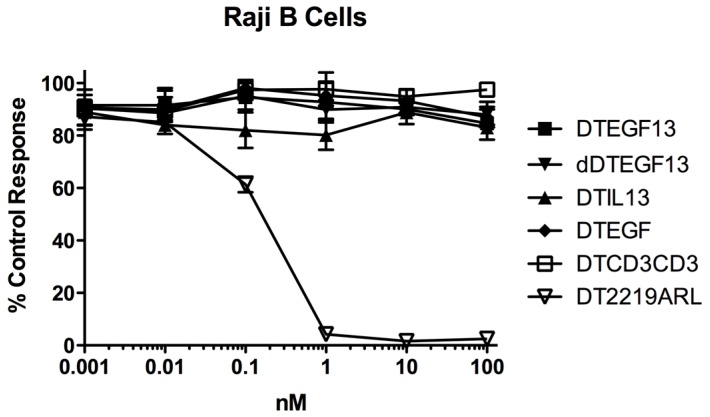
*In vitro* activity of dDTEGF13 against negative control Raji cells. To study the selectivity of dDTEGF13, EGFR^−^IL-13^−^ Raji cells (human Burkitts’ lymphoma cell line) were studied in a proliferation assay based on ^3^H thymidine uptake. No inhibition of proliferation was seen with DTEGF13, dDTEGF13, DTIL13, DTEGF and DTCD3CD3. However, inhibition did occur with control DT2219ARL because DT2219 consists of DT spliced to anti-CD19 and anti-CD22scFvs. Raji expresses both CD19 and CD22. These data indicate activity is mediated through the selective ligand binding.

As shown in Figure 3A the BLT is very potent with and IC50 of 0.019 nM on the EGF^+^IL-13^+^ cell line MDA-MB-231 (breast carcinoma cell line) and has greater activity than its monospecific counterparts in our *in vitro* proliferation screening assay. In addition, Figure 3B shows that dDTEGF13 has similar activity to the non-mutated parental control against the HT-29 human colon cancer carcinoma cell line. Figure 3C shows that the same was true when dDTEGF13 and non-muted parental was tested against the PC-3 prostate carcinoma. Figure 3D shows that the EGF13 ligand portion of dDTEGF13 molecule was mediated by its selective binding to target cells since dDTEGF13 was blockable with EGF13 ligand devoid of toxin against MiaPaCA-2 cells. To prove selective binding of the deimmunized drug, EGFR^−^IL-13^−^ Raji cells (human Burkitts’ lymphoma cell line) were studied in a proliferation assay based on ^3^H thymidine uptake (Figure 4). No inhibition of proliferation was seen with DTEGF13, dDTEGF13, DTIL13, DTEGF and DTCD3CD3. However, inhibition did occur with control DT2219ARL because DT2219ARL consists of DT spliced to anti-CD19 and anti-CD22scFvs. Raji expresses both CD19 and CD22 [22]. These data indicate activity is mediated through the selective binding of the ligands (Figure 4).

## 3. Evaluation of Immunogenicity

**Figure 5:**
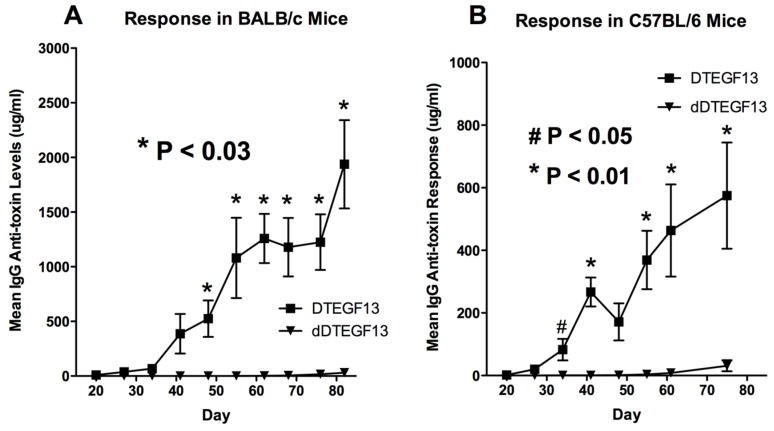
The immunogenicity of dDTEGF13. The immune response to deimmunized and non-mutated parental drug was determined by measuring anti-DT390 serum IgG on weekly samples of mice immunized with 0.25 µg of DTEGF13 (*n* = 5) or dDTEGF13 7 (*n* = 5). Measurements were made using an indirect ELISA and quantification of antibodies was determined using a standard curve generated with highly purified, high titer anti-DT antibody. Response was determined in BALB/c mice (* = *P* < 0.03) (**A**) and C57BL/6 mice (* = *P* < 0.01; **#** = *P* < 0.05) (**B**).

To determine whether DTEGF13 had been successfully deimmunized, groups of immunocompetent BALB/c mice (H-2^d^) were immunized weekly with 0.25 μg of either mutated dDTEGF13, or non-mutated parental DTEGF13. Animals were immunized i.p. over a period of 77 days. Serum samples were obtained weekly and analyzed using ELISA to detect anti-dDTEGF13 IgG. The results of the immunization experiment are summarized in Figure 5A and show statistical differences between the anti-toxin responses of the group of mice immunized with dDTEGF13 (*n* = 5) and the parental control (*n* = 5). After 12 immunizations on days 0, 1, 14, 21, 28, 35, 42, 49, 56, 63, 70, 77 and the last evaluation on day 84, the dDTEGF13 MC2 group showed minimal antibody response, while the parental group had an average anti-DT390 response of greater than 1500 μg/mL. Experiment ended after day 84 because an endpoint showing significant differences in immunization was achieved. In order to determine whether anti-toxin serum levels could be reduced in a second strain of mice that presents antigens differently, the same experiment was repeated with C57BL/6 mice (*n* = 5) with a different H-2 haplotype (H-2^b^). The results were nearly identical (Figure 5B).

### 3.1. Neutralizing Antibody

**Figure 6:**
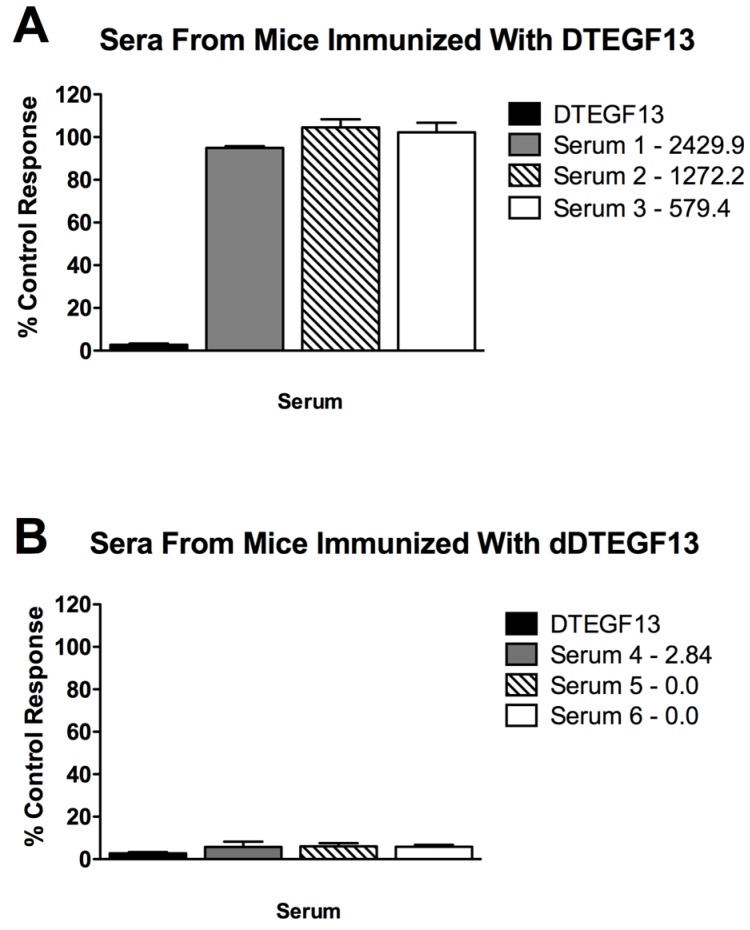
Neutralizing antibodies. Serum was collected from mice immunized with multiple injections of (**A**) parental DTEGF13 or (**B**) dDTEGF13 on day 56. Serum from individual mice was incubated with MiaPaCa-2 cells treated with a known inhibitory concentration of DTEGF13 in order to test for neutralization. Proliferation assays were performed by measuring tritiated thymidine uptake after 72 h. The serum concentration of IgG anti-toxin that was measured for each serum on day 56 is shown (μg/mL). There is a correlation between serum levels and the presence of neutralizing antibody.

To determine if neutralizing antibodies were present in the sera from immunized mice, sera from the mice in Figure 5A were added to a known inhibitory concentration 16 nM of dDTEGF13. The treated cells were then tested for tritiated thymidine uptake in proliferation assays. As shown in Figure 6A, the percent control response of treated MiaPaCa-2 cells and then the response of cells treated with day 56 serum from three different mice immunized multiple times with parental DTEGF13. High IgG serum anti-toxin levels in these mice (2,429, 1,272, and 579 μg/mL) correlated with high neutralizing activity. In contrast, Figure 6B shows the response of cells treated with day 56 serum from three different mice immunized multiple times with dDTEGF13. The low serum anti-toxin levels correlated with the lack of neutralizing activity. These data indicate that neutralizing antibodies were not present at day 56 in the serum of mice immunized with dDTEGF13, but were in serum of mice immunized with parental DTEGF13.

### 3.2. Efficacy of dDTEGF13 in an Intratumoral Nude Mouse Flank Tumor Model

**Figure 7:**
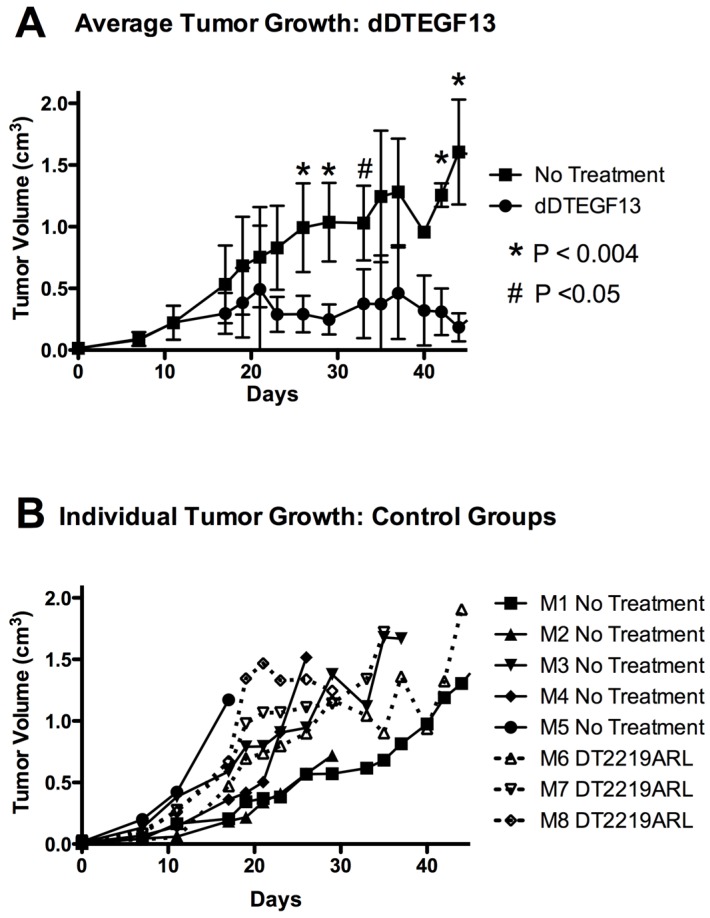
The effect of treatment of established PC-3 flank tumors with dDTEGF13. (**A**) Nude mice bearing PC-3 flank tumors were treated intratumorally with dDTEGF13, control DT2219ARL, or untreated. Tumors were treated with 8 injections of dDTEGF13. Average tumor volumes are shown for each treatment group (* = *P* < 0.004; **#** = *P* < 0.05); (**B**) Individual tumor volumes are shown for both the irrelevant control BLT treated group (DT2219ARL) and the untreated mice. The growth of individual tumors is plotted over time.

To test the ability of dDTEGF13 to inhibit tumor growth *in vivo*, PC-3 cells were injected into the flank of nude mice. Side-by-side testing of the deimmunized and parental DTEGF13 was not undertaken because of high levels of neutralizing antibodies in the parentially treated mice. Once the tumors were established and palpable, mice were treated with multiple intratumoral injections. dDTEGF13 was studied in a mouse model because the human EGF and IL-13 (of DTEGF13) reacts with mouse EGFR and IL13R, respectively. Figure 7A shows mean tumor volume data in which groups of mice were given injections (0.25 μg/injection) with dDTEGF13 (*n* = 6) (starting on day 15 until 25) or as control group with either no treatment (*n* = 6) or treatment with an irrelevant immunotoxin control, DT2219ARL (*n* = 3). Despite the fact that dDTEGF13 therapy was terminated on day 25, tumor growth remained inhibited until day 49 indicating that continuous exposure to the drug is not mandated for efficacy. The experiment was terminated according to the rules of the institutional review board when tumor size achieved >1 cm^3^. Figure 7B shows tumor growth of individual control mice. The multiple injections of dDTEGF13 were effective at preventing tumor growth compared to the negative control.

## 4. Discussion

The contribution of this work is the first-time reporting of deimmunized diphtheria toxin that may now be used in molecular drug development. Also, the method of deimmunization is important since it took advantage of eliminating only seven highly hydrophilic amino acids that play a major role in the generation of anti-B cell responses and was much less time consuming and cost-effective than epitope mapping techniques.

We undertook the study knowing that we would need to identify which amino acids to change, where to change them, and how many to change. An established paradigm in protein folding is that proteins tend to arrange themselves in asymmetric patterns with the polar side chains extending out into the solvent and apolar side chains buried within the protein [23]. Several protein structural antigenic prediction algorithms are based on this principle, including the Hopp and Woods [24] and the Jameson and Wolf method [25]. In fact, Hopp and Woods assigns R, K, D, E, and Q as amino acids with the highest hydrophilicity index values [26]. Other investigators are aware of the immunogenic nature of these side chains [27] and PE was partially deimmunized by mutating combinations of only these residues [7]. Thus, using the known X-ray crystallographic structure of DT390, we identified 24 potential amino acids (R, K, D, E, and Q) located in prominent positions on the molecular surface. We mutated DT390 toxin located on our DTEGF13 molecule. The DTEGF13 parental construct was mutated simultaneously with three site-specific PCR primers to generate triple mutants. Those that did not undergo activity loss were further mutated finally resulting in one construct with 7 critical point mutations, but with minimal activity loss. Mutating DTEGF13 and expressing the product permitted us to effectively screen the mutants for activity *in vitro* anti-carcinoma assays compared to the non-muted parental form.

How many epitopes would be sufficient for deimmunzation? No such information is currently available for DT, but there is evidence for other biological molecules. For example, the study of horse cytochrome C by Jemmerson (14 kDa) revealed three major non-overlapping epitope groups [28]. Another example is three major non-overlapping epitope groups identified for lysozyme [29]. Seven major immunogenic epitopes have been identified for PE, which is a close cousin of DT with an identical mechanism of action [7]. These immunogenic epitopes were not diffusely distributed, but were located at specific positions. Based on these findings, it is not unreasonable to speculate that DT390 may also have at least seven immunogenic epitopes in positions scattered across the molecule. Thus, we mutated DT in seven different unrelated positions in two different mutants that we constructed.

It was necessary to determine which were the best amino acids to target for point mutation. Because DT has been studied for many years, the structure and mechanism is well established, so all of our point mutations that we chose were located well away from the active site. A 3D pymol molecular model was constructed from the X-ray crystallographic structure of DT and residues were selected based on prominent cell surface positions that would likely provide accessibility to high affinity antibody recognition. Other factors were considered. For example, Lambotte and colleagues identified a hydrophilic surface lipid-associating domain (SLAD) composed of two hydrophilic alpha helices (residues 210–227 and residues 237–248) [30]. These may play a role in the process by which the A fragment is translocated suggesting a prominent membrane interactive position. Thus, residues 227 and 225 were targeted for mutation. Additionally, DT activation requires protease nicking at an arginine rich region located in a disulfide loop [31]. The loss of amino shifts amino acid 184 to a prominent cell surface rendering it a potential target. None of the amino acids that we targeted were located in molecular crypts that would render immunological recognition difficult.

In order to address whether point mutation removed T cell or B cell epitopes, we immunized two different strains of mice with different MHC haplotypes (H-2b and H-2d). MHC molecules are supposed to load different regions of peptide fragments for presentation as T cell epitopes and T and B epitopes are not necessarily linked [32,33]. Our mutated drug was equally as effective in reducing anti-toxin responses in both strains, indicating that B cell epitopes rather than T cell epitopes have been eliminated from DT.

## 5. Experimental Section

### 5.1. Construction of dDTEGF13

The DTEGF13 gene was originally synthesized using assembly PCR [11]. In its final configuration, gene (from 5′ end to 3′ end) consisted of an Nco1 restriction site, an ATG initiation codon, the first 390 amino acids of the DT molecule (DT390), the 7 amino acid EASGGPE linker, the genes for human EGF and IL-13 linked by a 20 amino acid segment of human muscle aldolase (hma), and a XhoI restriction site (Figure 1A). The final 1755bp NcoI/XhoI target gene was spliced into the pET21d expression vector under control of an isopropyl-*b*-d-thiogalactopyranoside (IPTG) inducible T7 promoter. DNA analysis was used to verify that the gene was in correct sequence (Biomedical Genomics Center, University of Minnesota, Minneapolis, MN, USA). DTCD3CD3 was synthesized as a control by fusing two repeating scFvs recognizing human CD3epsilon to DT390 [34].

To create a deimmunized drug, DTEGF13 was mutated using the QuickChange Site-Directed Mutagenesis Kit (Stratagene, La Jolla, CA, USA) and site-specific mutations were confirmed by DNA sequencing.

### 5.2. Isolation of Inclusion Bodies, Refolding and Purification

These procedures were previously described [34]. Plasmids were transformed into Escherichia coli strain BL21 (DE3) (Novagen, Madison, WI, USA). Following overnight culture, bacteria were grown in Luria broth. Gene expression was induced with the addition of IPTG (FischerBiotech, Fair Lawn, NJ, USA). Two hours after induction, bacteria were harvested by centrifugation. Cell pellets were suspended and homogenized. Following sonication and centrifugation, the pellets were extracted and washed. Inclusion bodies were dissolved and protein refolded. Refolded proteins were purified by ion exchange chromatography (Q sepharose Fast Flow, Sigma-Aldrich, St. Louis, MO, USA) using a continuous gradient.

### 5.3. Antibodies and Cells

Anti-Ly5.2, a rat IgG2a from clone A20-1.7 was generously provided by Dr. Uli Hammerling, Sloan Kettering Cancer Research Center, New York, NY, USA. Anti-Ly5.2 was used as a control since it recognized mouse CD45.1, a hematopoietic cell surface marker not expressed on human cells.

### 5.4. Human Cell Lines

The human prostate cancer cell line PC-3 [35], human colorectal cell line HT-29 [36], the human pancreatic carcinoma cell line MiaPaCa-2 [37], and the Burkitt’s Lymphoma cell line Raji were obtained from American Type Culture Collection (ATCC, Rockville, MD, USA). Cells were maintained in RPMI-1640 media (Cambrex, East Rutherford, NJ, USA) supplemented with 10% fetal bovine serum, 2 mmol/L L-glutamine, 100 units/mL penicillin, and 100 μg/mL streptomycin. All carcinoma cells were grown as monolayers and Raji cells in suspension using culture flasks. Cell cultures were incubated in a humidified 37 °C atmosphere containing 5% CO_2_. When adherent cells were 80%–90% confluent, they were passaged using trypsin-EDTA for detachment. Only cells with viability >95%, as determined by trypan blue exclusion, were used for experiments.

### 5.5. Proliferation Assay

To determine the effect of drug on tumor cells, cells (2 × 10^4^) were plated in a 96-well flat-bottom plate in RPMI supplemented with 10% fetal bovine serum, 2 mM *l*-glutamine, 100 U/mL penicillin, 100 μg/mL streptomycin. BLT in varying concentrations was added to triplicate wells containing cells [38]. The plates were incubated at 37 °C, 5% CO_2_ for 72 h. Cells were then incubated with one µCi [methyl-3H]-thymidine (GE Healthcare, Little Chalfont, Buckinghamshire, UK) per well for eight hours and harvested onto glass fiber filters, washed, dried and counted for ten minutes in a standard scintillation counter. Data were analyzed using Prism 4 (GraphPad Software, Inc., La Jolla, CA, USA) and were presented as “percent control response” calculated by dividing the counts per minute (CPM) of untreated cells by the CPM of the immunotoxin-treated cells (×100).

Blocking studies were conducted to test specificity. Briefly, 1, 10, 100 or 1000 nM EGF13 devoid of toxin were added to media containing 0.001, 0.01, 0.1, 1, 10 or 100 nM DTEGF13 [39]. Resulting mixtures were added to wells containing tumor cells and proliferation was measured by 3H-thymidine uptake as described. Data were presented as percent control response.

### 5.6. Detecting Neutralizing Antibodies

To detect neutralizing antibodies, 90% serum from immunized mice was added to cells treated with a known inhibitory concentration of DTEGF13 (16 nM). Proliferation assays were then carried out as described above. For detection of serum IgG anti-toxin content ELISA Assay was used.

### 5.7. Detecting Anti-toxin Antibodies

Our assay to detect IgG anti-toxin antibodies was previously reported [40]. Briefly, immunocompetent normal BALB/c mice (NCI) were immunized with weekly injections of 0.25 µg non-mutated DTEGF13 or mutated DTEGF13 7 mut (dDTEGF13). After 5 injections, serum was collected 4 days after the final injection. A standard ELISA assay was used in which recombinant DT390 was adhered to the plate. Test serum from the immunized mice was then added followed by the detection antibody, anti-mouse IgG peroxidase (Sigma-Aldrich, St. Louis, MO, USA). Plates were developed with *o*-Phenylenediamine Dihydrochloride (Pierce Biotechnology, Rockford, IL, USA) for 15 min at room temperature. The reaction was stopped with the addition of 2.5 M H_2_SO_4_. Absorbance was read at 490 nm and the final concentration was determined from a standard curve using highly purified anti-DT390. All samples and standards were tested in triplicate.

### 5.8. In vivo Efficacy Studies

Male nu/nu mice were purchased from the National Cancer Institute, Frederick Cancer Research and Development Center, Animal Production Area and housed in an Association for Assessment and Accreditation of Laboratory Animal Care-accredited specific pathogen-free facility under the care of the Department of Research Animal Resources, University of Minnesota. Animal research protocol number 1306-30718A was approved by the University of Minnesota Institutional Animal Care and Use Committee. All animals were housed in microisolator cages to minimize the potential of contaminating virus transmission.

Our flank tumor model was previously reported [11]. We choose this route of administration because the major point of this experiment was not to mimic a clinical protocol, but was to determine if deimmunized DTEGF13 was efficacious. Direct intratumoral administration circumvents the major problems of systemic administration. These include inefficient distribution to tumor site related to the distance traveled and unfavorable tumor vasculature dynamics (high interstitial pressures) [41]. Consequently, intratumoral therapy guarantees more consistent and reliable delivery of recombinant toxin fusion proteins to the targeted site with limited systemic exposure, which maximizes efficacy. For induction of tumor mice were injected in the left flank with 4 × 10^6^ PC-3 cells suspended in 100 μL of a 1:1 RPMI/Matri-Gel mixture. Once palpable tumors had formed (day 15), mice were divided into groups and treated with multiple injections of DTEGF13 from day 15 to day 25. All BLT were administered by intratumoral injection using 3/10 cc syringes with 29 gauge needles. All treatments were given in a 100-μL volume of sterile PBS. Tumor size was measured using a digital caliper, and volume was determined as a product of length, width, and height. The experiment were terminated according to the faculties animal board when tumor size achieved >1 cm^3^ and for all animals on day 49 by achieving the endpoint, showing significant treatment advantages compared to control group.

## 6. Conclusions

In summary, we have assembled a potent anti-carcinoma agent in dDTEGF13. Studies indicate a precipitous decline in its ability to generate anti-toxin antibodies in immunocompetent mice due to B cell epitopic mutations in the toxin moiety. The drug is effective against pancreatic, breast cancer, and prostate cancer cell lines. Assuming that mouse is a model of DT immunogenicity in the same way that PE is a model of immunogenicity, we may at last have found a means to reduce the production of anti-DT antibodies in human genetically engineered biologicals. Furthermore, this technique is much less costly than other techniques of deimmunization, and it may be applied to other immunogenic biologicals as well.
